# Cysts mark the early stage of metastatic tumor development in non-small cell lung cancer

**DOI:** 10.18632/oncotarget.23785

**Published:** 2017-12-30

**Authors:** Chitra Thakur, Ulf R. Rapp, Thomas Rudel

**Affiliations:** ^1^ Cancer Metastasis Group, Department of Molecular Biology, Max Planck Institute of Biochemistry, Martinsried 82152, Germany; ^2^ Department of Pharmaceutical Sciences, Eugene Applebaum College of Pharmacy and Health Sciences, Wayne State University, Detroit, Michigan 48201, USA; ^3^ Department of Lung Development and Remodeling, Max Planck Institute for Heart and Lung Research, Member of the German Center for Lung Research (DZL), Bad Nauheim 61231, Germany; ^4^ Department of Microbiology, Biocenter, University of Wuerzburg, Wuerzburg D-97074, Germany

**Keywords:** lung cancer, metastasis, cysts, lineage tracing, dedifferentiation

## Abstract

Identifying metastatic tumor growth at an early stage has been one of the biggest challenges in the treatment of lung cancer. By genetic lineage tracing approach in a conditional model of Non-Small Cell Lung Cancer (NSCLC) in mice, we demonstrate that cystic lesions represent an early stage of metastatic invasion. We generated a mouse model for NSCLC which incorporated a heritable DsRed fluorescent tag driven by the ubiquitous CAG promoter in the alveolar type II cells of the lung. We found early cystic lesions in a secondary organ (liver) that lacked the expression of *bona fide* lung makers namely Scgb1a1 and surfactant protein C Sftpc and were DsRed positive hence identifying lung as their source of origin. This demonstrates the significant potential of alveolar type II cells in orchestrating the process of metastasis, rendering it as one of the target cell types of the lung of therapeutic importance in human NSCLC.

## INTRODUCTION

Metastases of cancer cells from a primary tumor to distant sites are responsible for ninety percent of human cancer deaths, thereby contributing to one of the major causes of cancer mortality [1]. Lung cancer is the leading cause of cancer deaths worldwide [2]. Non-small cell lung cancer (NSCLC) being the most dangerous cancer in terms of mortality rates, commonly metastasizes to brain, liver, and bone. The most promising and effective approach to improving patient’s survival is by the early detection of primary tumor and metastasis events. However, our current knowledge in the field has been insufficient in effectively tackling the problem of early diagnosis of cancer. For many patients by the time cancer is detected, metastasis has already occurred. Furthermore, several studies including our own have indicated metastasis to be a very early phenomenon occurring concomitant with the primary tumor development or even prior to the formation of an identifiable primary tumor [3].The biggest challenge in identifying early disseminating tumor cells is the unavailability of reliable markers that can distinguish circulating cancer cells from normal cells either in the blood stream or at the sites of secondary organs [4]. RAS-RAF-MEK-ERK MAPK signal transduction pathway has been implicated in many lung cancers, with activating mutations in K-Ras oncogene being the most common oncogenic alterations in lung adenocarcinoma [5–7]. Recent studies show C-RAF rather than B-RAF as an essential factor in the development of K-Ras driven NSCLC [8] and RAF/MYC cooperation in tumorigenesis has been well elaborated [9]. Among different transcription factors, MYC is the most frequently amplified oncogene that promotes tumorigenesis in a wide range of tissues [10–12]. MYC amplification occurs in 15% to 30% SCLCs and 5% to 10% NSCLCs [13]. We have previously shown the ability of c-MYC to induce metastasis in a C-RAF driven mouse model for NSCLC [3]. Additionally, we consistently detected small lesions that appeared alien to the secondary organ and the identity of these lesions could not be reliably established. In the present report, using the Tet O with Cre/lox P system of gene induction we established a model that carried c-MYC and C-RAF as the driver oncogenes; resulting in the generation of quadra (SPCrtTA/TetO-Cre/SPC-c-MYC/DsRed) and penta (SPCrtTA/TetO-Cre/TetO-C-RAF BxB/SPC-c-MYC/DsRed) transgenic reporter mice. Our study revealed that the lesions are derived from alveolar type II cells and are indeed indicative of the initial stage of metastasis. Co-occurrence of DsRed positive cystic and papillary tumor lesions in the same organ suggested periodic migration of metastasizing cancer cells. Our findings thus identified the origin of cystic lesions frequently observed in liver metastases and established them as early events of cancer metastasis. We found that the early events of cancer metastasis require reprogramming to a less differentiated progenitor state and is suggestive of a prerequisite for an invasion to secondary organs by tumor cells. This is indicated by the fact that the loss of differentiation markers like Sftpc and Scgb1a1 and/or dedifferentiation of cancer cells occur at some point during the process of malignant progression. This is the first report of genetic lineage tracing of metastasis in NSCLC demonstrating the contribution of alveolar type II pneumocytes in lung neoplasias and metastasis.

## RESULTS

### Generation of reporter transgenic mouse lines for lineage tracing

**Figure 1:**
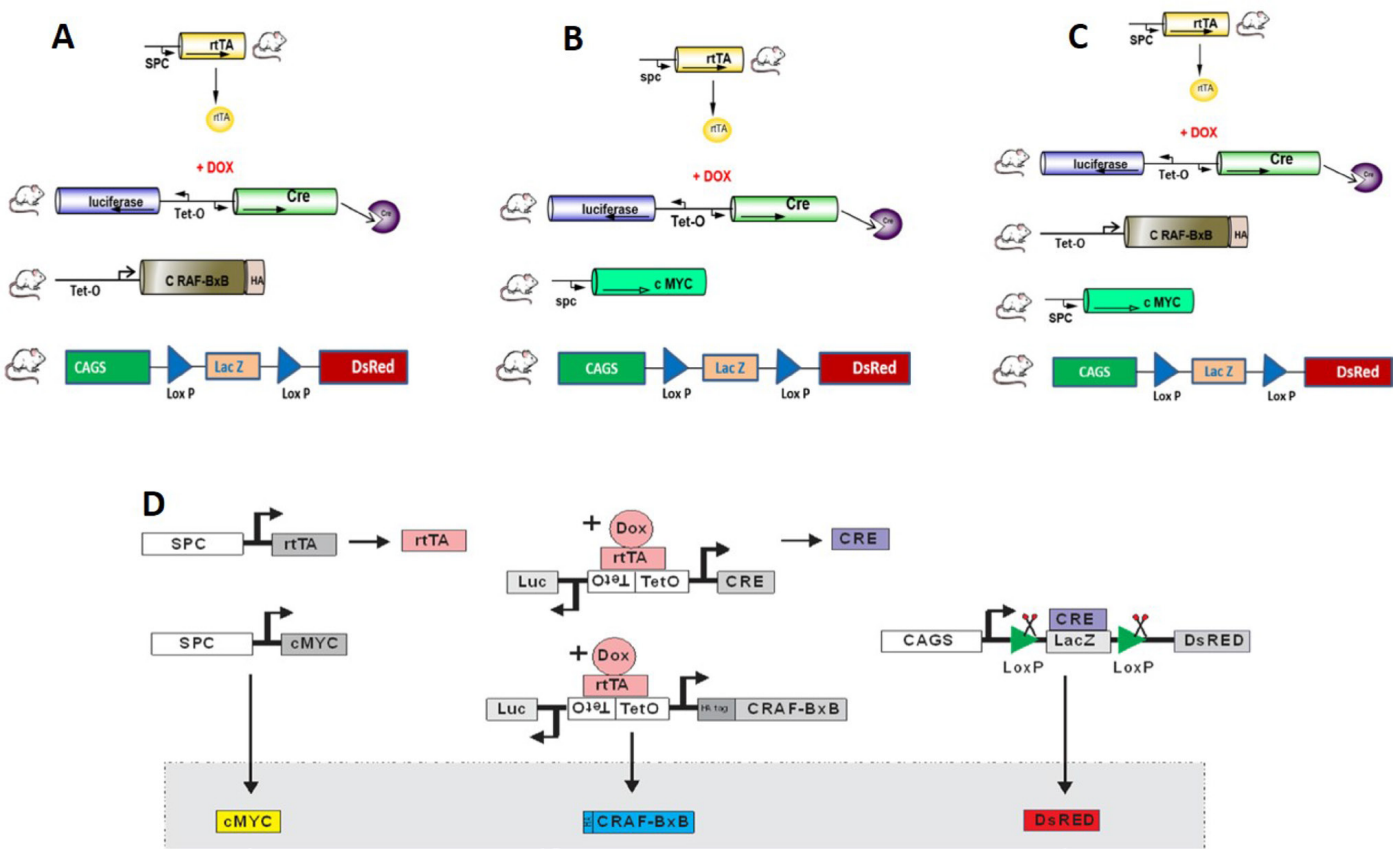
Reporter transgenic mice lines generated for lineage tracing of alveolar type II cells in a murine model of NSCLC Constitutive active C-RAF (C-RAF BxB) and c-MYC has been incorporated with the reporter LacZ/DsRed, under the control of human SPC promoter resulting in the non-metastatic model of quadra transgenic (**A**) SPCrtTA/TetO-Cre /TetO-C-RAF BxB/DsRed (hereafter C-RAF BxB-DsRed) and a metastatic model (**B**) SPCrtTA/TetO-Cre /SPC-c-MYC/DsRed (hereafter MYC-DsRed) respectively. Combining c-MYC and C-RAF BxB with the reporter Lac Z/DsRed resulted in the penta transgenic (**C**) SPCrtTA/TetO-Cre /TetO-C-RAF BxB/SPC c-MYC/ DsRed (hereafter MYC-BxB-DsRed) which is also a metastatic model for NSCLC. Induction with doxycycline results in the expression of the lineage tag DsRed specifically in alveolar type II cells. (**D**) Schematic representation of the genetic lineage tracing in a metastatic model. Number of animals generated (n), *n* = 62 C-RAF BxB-DsRed (A), *n* =52 MYC-DsRed (B) and *n* = 19 MYC-BxB-DsRed (C).

To select a suitable tag that can be monitored easily in live cells, in tissues and which can be exploited for imaging purpose, a reporter mouse was chosen. The Lac Z/DsRed transgenic mice express beta-galactosidase (*lacZ*) under the control of the chicken beta actin promoter coupled with the cytomegalovirus (CMV) immediate early enhancer. When crossed with a Cre recombinase-expressing strain, *lacZ* expression is replaced with red fluorescent protein (DsRed*MST) expression in tissues expressing Cre recombinase. We utilized transgenic mice in which the human SPC (Sftpc) gene promoter is used to express the reverse tetracycline transactivator (rtTA) thus placing the expression of Cre-recombinase (CRE) under the conditional control of doxycycline. Expression of Cre was used to permanently label cells with Red fluorescent protein (DsRed) in alveolar type II cells. Distinct lines of transgenic mice that express rtTA under the control of the human surfactant-associated protein C (Sftpc/SPC) gene promoter were bred to TetO-Cre mice and reporter mice (LacZ/DsRed) creating triple transgenic mice as SPCrtTA/TetO-Cre /DsRed (here in designated as DsRed). Once we obtained triple transgenic reporter mice, multiple rounds of successive breeding with the oncogenic mice gave rise to Quadra as SPCrtTA/TetO-Cre /SPC-c-MYC/DsRed (Figure 1B here in designated as MYC-DsRed) and penta transgenic as SPCrtTA/TetO-Cre/TetO-C-RAF BxB/SPC-c-MYC/DsRed (Figure 1C here in designated as MYC-BxB-DsRed). We also established quadra transgenic with an inducible C-RAF and the reporter DsRed alone SPCrtTA/TetO-Cre /TetO-C-RAF BxB/DsRed (Figure 1A here in designated as C-RAF BxB-DsRed) as control lines for metastasis experiments. The schematic representation of the genetic lineage tracing of alveolar type II cells in a metastatic model has been depicted in Figure 1D. The rationale behind choosing the C-RAF, c-MYC and C-RAF/MYC combination comes from the observation that has been reported in our previous studies [3, 14]. C-RAF BxB transgene expressed in alveolar type II cells induces the development of premalignant adenomas. This was the first classical mouse model for human NSCLC employing the RAF gene [14]. Mice bearing C-RAF adenomas showed the presence of micro-metastasis in lymph nodes but failed to show macro-metastasis in the distant organs.

With the c-MYC transgene, tumors developed late with incomplete penetrance but macroscopic liver metastasis was readily observed. However, in the MYC/RAF BxB mice, metastasis developed earlier and with higher incidence. C-RAF and c-MYC cooperate to accelerate the lung tumor formation and form distant metastasis in liver [3].

### Genetic labeling marks alveolar type II cells and tumor cells with DsRed in the lungs of the transgenic reporter mice

**Figure 2:**
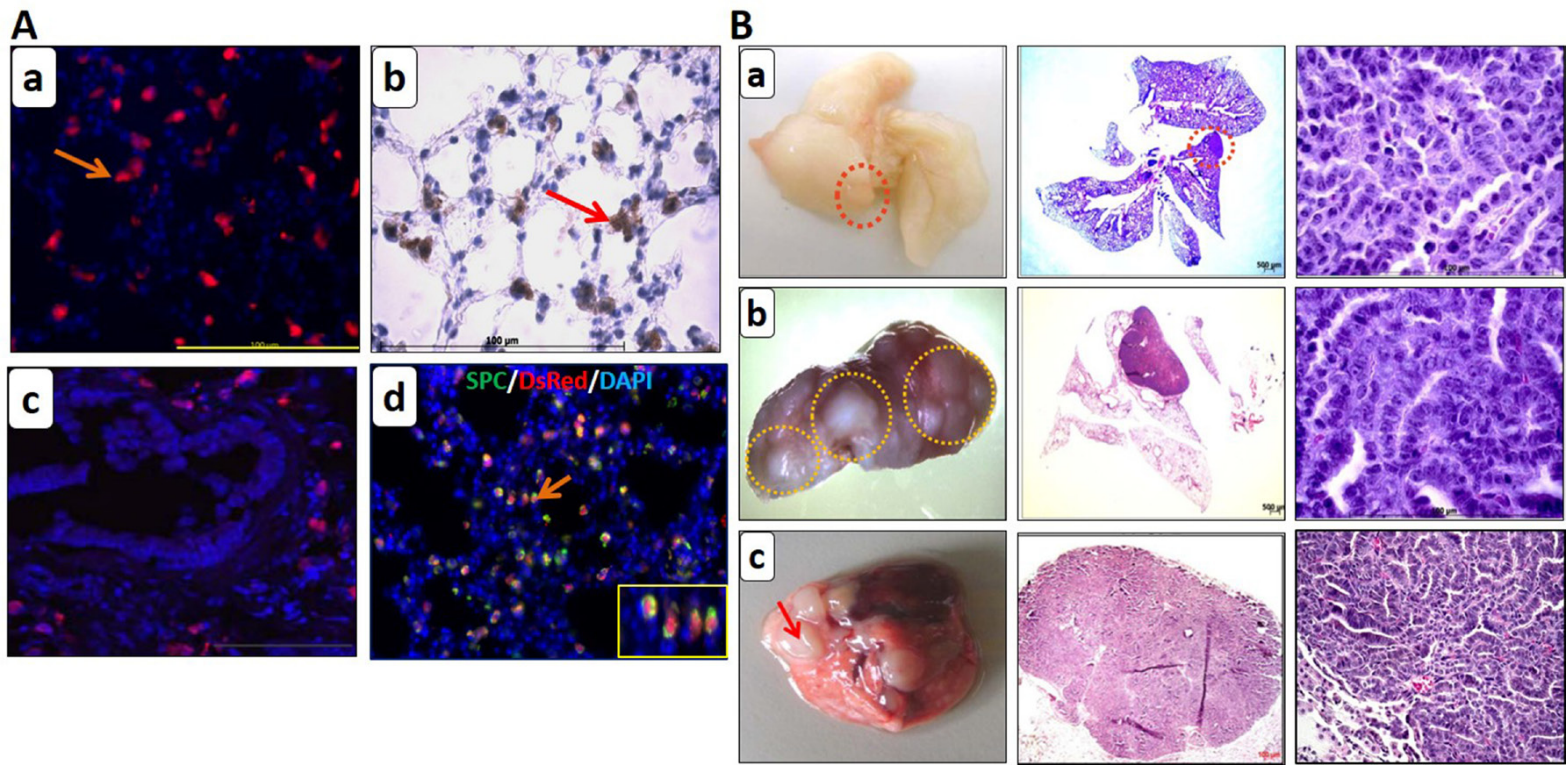
Genetic lineage labeling marks alveolar type II cells with DsRed in the non-neoplastic lungs while tumors were generated in the neoplastic lungs of the reporter transgenic mice (**A**) DsRed immuno-staining on the (a) frozen and (b) paraffin lung sections of the induced non-neoplastic DsRed transgenic mice depicting DsRed cells (red and brown resp.). (c) DsRed is expressed in the alveolar type II cells only as columnar Clara cells in the bronchioles are negative for the DsRed expression. (d) Double immuno-fluorescence staining for SPC and DsRed on frozen lung sections display the co-localization of SPC cells (green) with DsRed (red). The inset shows the double positive cells in high magnification (40X). Representative images from five mice, scale bar = 100 μm. (**B**) Induction of neoplastic transgenic reporter mice drives/promotes tumor formation in the lung. (a) C-RAF BxB-DsRed transgenic mice (9 to 12 months induced, *n =* 3), (b) MYC-DsRed (12 to 15 months induced, *n =* 2) and (c) MYC-BxB-DsRed transgenic mice (12 months induced, *n =* 2) were analyzed macroscopically and representative images of the whole lung were shown (left panels, tumors encircled in red). H&E staining of lung sections for tumor structure (middle panels, scale bar = 500 μm) and at higher magnification for cell morphology (right panels, scale bar = 100 μm).

Once the transgenic lines were established, the first step was to check the robustness of our labeling system. For that purpose, induced non-neoplastic triple transgenic DsRed mice (SPCrtTA/TetO-Cre/DsRed) were analyzed for DsRed expression. DsRed staining revealed many distinct cells positive for DsRed [Figure 2A (a) and (b)]. No DsRed cells were detected in the bronchioles [Figure 2A (c)]. This suggests that the labeling specifically marked only alveolar type II cells whereas the Clara cells comprising the bronchioles are rendered negative. Double staining showed co-localization of DsRed cells with SPC (Sftpc) positive cells in the airway epithelium [Figure 2A (d)], confirming that the switched cells are indeed alveolar type II cells. These switched cells are the ones that were turned ON for DsRed expression upon Cre activation when animals are doxycycline induced.

**Figure 3:**
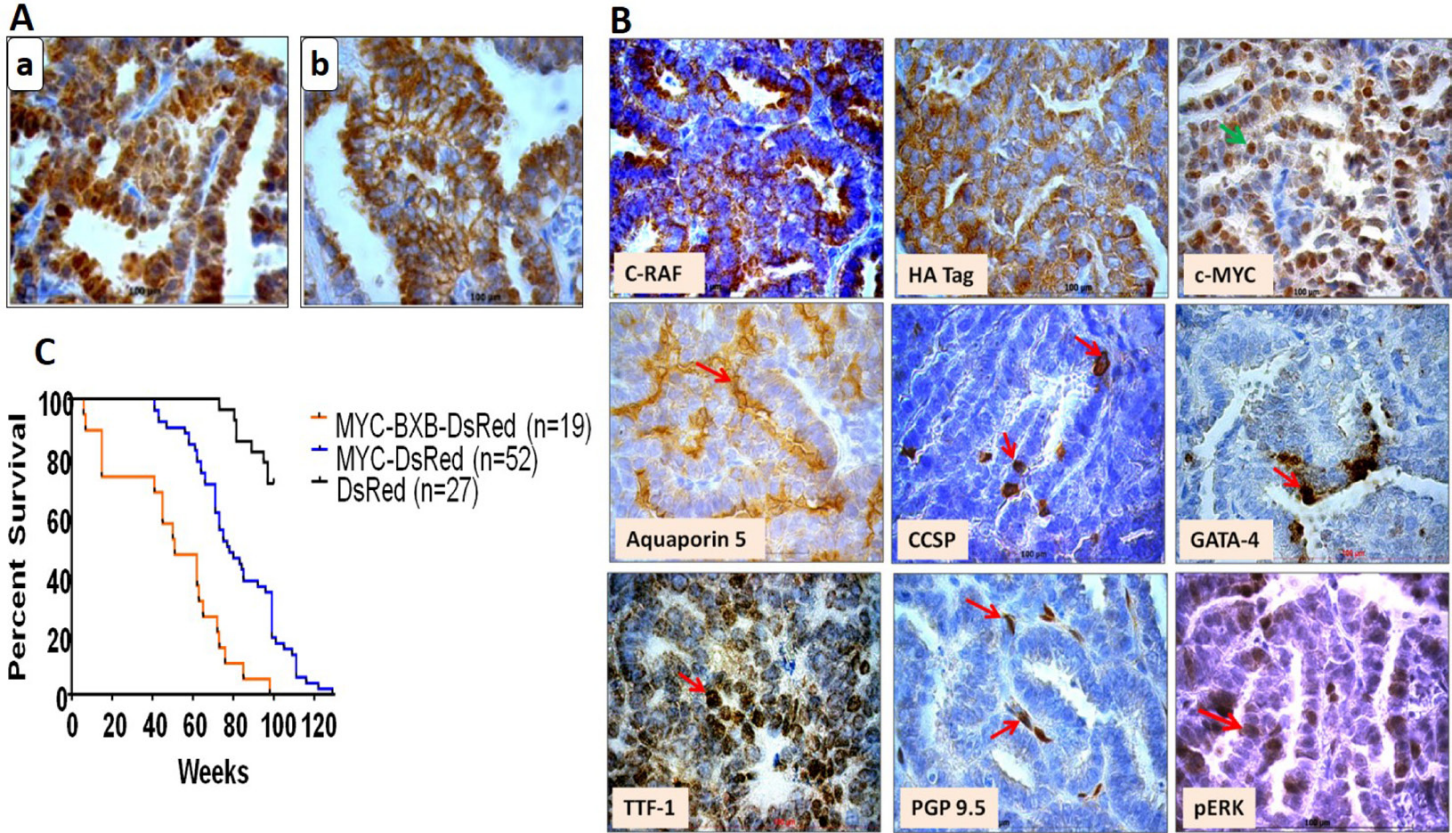
Primary lung tumors from the neoplastic transgenic reporter mice displayed DsRed expression (**A**) Lung tumors from the metastatic model, MYC-BxB-DsRed mice (>12 months) were labeled with the lineage tag DsRed and express alveolar type II cell marker, Sftpc. Immunohistochemical staining of the paraffin-embedded lung sections displayed nucleo-cytoplasmic expression of DsRed (a) and membranous localization of Sftpc (b). Scale bar = 100 μm. *n =* 2. (**B**) Immunohistochemical staining of the tumors obtained from MYC-BxB-DsRed mice (>12 months) for lung differentiation markers. Paraffin-embedded tumor-bearing lungs were immunostained for the indicated markers. Scale bar =100 μm. *n =* 2. (**C**) Kaplan-Meier plot for percent survival of labeled compound mice as for DsRed (*n =* 27), MYC-DsRed (*n* = 52), and MYC-BxB-DsRed (*n* = 19) mice. Log-rank (Mantel-Cox) comparison for percent survival for compound mice MYC-BxB-DsRed versus DsRed mice = *p* > 0.0001; labeled MYC-DsRed mice versus DsRed mice = *p* < 0.0001. *n =* number of animals. The median survival of MYC-BxB-DsRed is 51 weeks and for MYC-DsRed is 77.5 weeks.

Since c-MYC is known to be a metastatic gene for NSCLC and induced macro-metastasis in the liver; next step was to study the involvement of alveolar type II cells in lung tumor metastasis. For this purpose, C-RAF BxB-DsRed, MYC-DsRed, and MYC-BxB-DsRed transgenic mice were induced with doxycycline food and were analyzed after a period of one year. Multiple macroscopic lung tumors were formed in MYC-DsRed [Figure 2B (b)] and MYC-BxB-DsRed [Figure 2B (c)] mice. Whereas tumors derived from the MYC-DsRed mice exhibited columnar cell phenotype [Figure 2B (b)]; tumors obtained from the MYC-BxB-DsRed mice displayed a mixed phenotype comprising both cuboidal and columnar cell structure [Figure 2B (c)]. Interestingly, tumors from the C-RAF BxB-DsRed mice consists of only cuboidal cells [Figure 2B (a)]. This indicates that introduction of c-MYC induces the tumor cells to gain a new phenotype, i.e. the columnar structure, which appears to be more malignant and hence a signature for the appearance of papillary carcinomas in the advanced stages of tumor progression. Subsequently, lung tumors from MYC-BxB-DsRed mice (>12 months) were analyzed for the expression of the cell type-specific markers Sftpc and DsRed, along with the driver oncogenes fueling the tumors, i.e. C-RAF and c-MYC. Heterogeneous expression of DsRed persisted within the tumors [Figure 3A (a)] which were subsequently confirmed by the similar Sftpc expression pattern [Figure 3A (b)]. Membranous C-RAF and HA tag along with strong nuclear c-MYC and TTF-1 were detected in the tumor cells. Marker of alveolar type I cell lineage aquaporin 5 and heterogeneous expression of nuclear pERK were detected in the tumor. Additionally, PGP 9.5, a marker for immature neuroendocrine cells [15] as well as a candidate tumor marker for NSCLC [16], were expressed in the tumor (Figure 3B). Survival analysis of non-neoplastic DsRed, and neoplastic MYC-DsRed and MYC-BxB-DsRed mice showed accelerated death in MYC-BxB-DsRed mice (Figure 3C).

### Labeling of small cryptic lesions with DsRed in the lungs of the non-metastatic C-RAF BxB-DsRed mice

**Figure 4:**
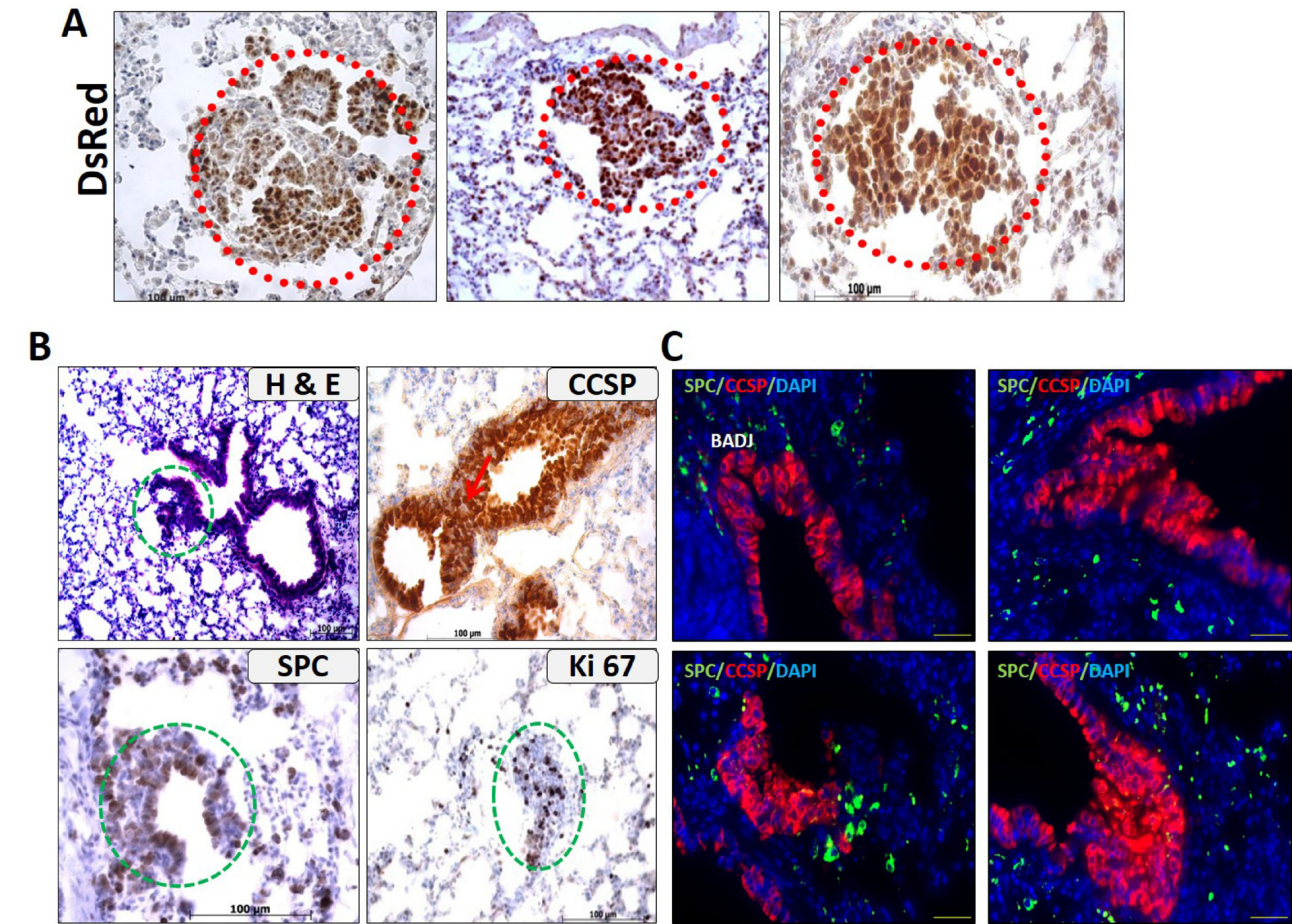
Lungs of the non-metastatic C-RAF BxB-DsRed mice displayed cryptic lesions and hyperplasias (**A**) Small cryptic adenoma-like lesions are labeled with DsRed in the C-RAF BxB-DsRed lungs (indicated by red circles). (**B**) The lungs from these mice also showed abundant hyperplasias (H&E) that are positive for CCSP, SPC and are proliferating as seen by the Ki 67 staining (indicated by green circles). (**C**) Co-Immunofluorescence staining for SPC and CCSP on these lungs showed the absence of double positive cells. The image is shown especially for the BADJ regions where BASC are expected to be seen. (BADJ = Bronchio-alveolar duct junction). Paraffin-embedded lung sections stained for the indicated markers. Scale bar = 100 μm. Representative images from 10 animals of 6 weeks to 4 months of age post induction.

One of the important features that were readily observed in the C-RAF BxB-DsRed mice is the presence of a small cryptic cluster of cells. Notably, these lesions appear quite early, much before the onset of frank visible tumors. In an attempt to ascertain the identity of these cryptic lesions, we immunostained the paraffin-embedded lung sections of the C-RAF BxB-DsRed mice with the lineage marker DsRed. We detected many DsRed positive cells within the lesions suggesting that they are possibly the early tumor lesions which will eventually progress to visible macroscopic adenomas (Figure 4A). Another striking feature of the lungs of these transgenic mice was the presence of multiple bronchiolar hyperplasic regions [Figure 4B (H&E)]. Most of the hyperplasias were continuous with the alveolar space adjacent to the respiratory bronchioles. To unravel the origin of these hyperplasias, we immunostained the paraffin-embedded lung sections with the SPC and CCSP antibodies for the specific alveolar and bronchiolar cell determination. The isolated hyperplasias were all positive for CCSP, suggesting that these lesions arose from the Clara cell and its precursors. They also showed some SPC expression and are proliferating as we observed many Ki 67 positive cells in the hyperplasic regions (Figure 4B). Also, to look for possible BASC at the BADJ (Bronchioalveolar duct junction) regions of these hyperplasias, we performed double immunofluorescence staining with the SPC and CCSP antibodies. We did not detect any BASC cells in the hyperplasias (Figure 4C).

### Premalignant pleomorphic cell clusters are labeled with DsRed and are suggestive of the metastatic initiating cells

**Figure 5:**
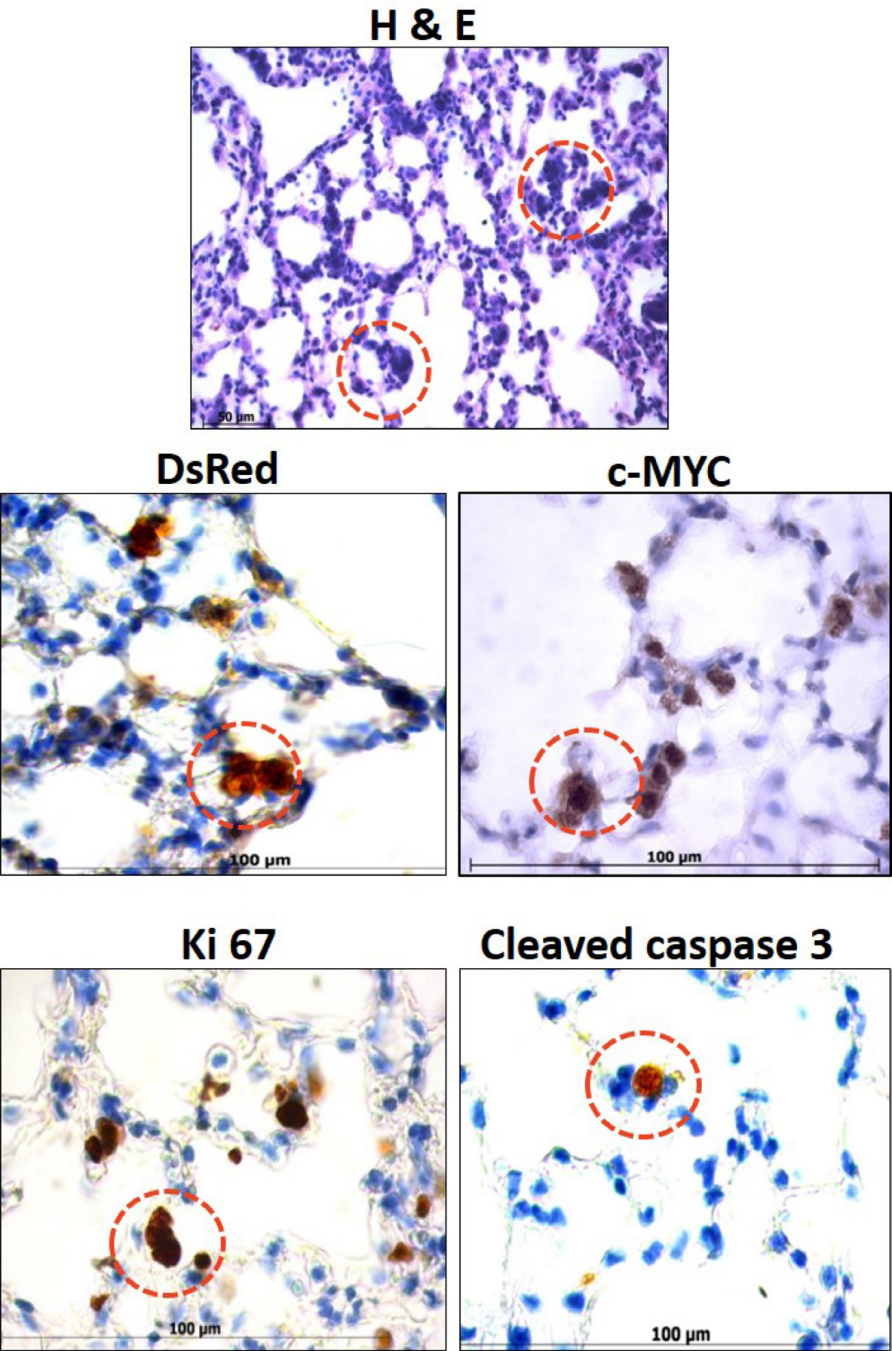
Occurrence of unique premalignant lesions in the lungs of metastatic transgenic reporter mice Representative images from MYC-DsRed and MYC-BxB-DsRed transgenic lungs (>12 months) displayed the presence of unique premalignant lesions composed of the pleomorphic cluster of cells that are labeled with DsRed and express c-MYC which are in a constant status of proliferation and apoptosis. Paraffin-embedded lung sections stained with H&E shows the pleomorphic clusters (in red circles) which are immuno-positive for the indicated markers DsRed, c-MYC, Ki 67 and cleaved caspase 3 (brown cells within red circles). Scale bar = 100 μm. Representative images from five animals that belonged to MYC-DsRed and MYC-BxB-DsRed transgenic mice.

Lungs of the metastatic transgenic mice model MYC-DsRed and MYC-BxB-DsRed showed the presence of a unique group of cells. Interestingly, this unique group of cells appears as clusters of pleomorphic cells and is detected only in the lungs of MYC-DsRed and MYC-BxB-DsRed mice but not in the C-RAF BxB-DsRed lungs. They are speculated to be the potential sources for metastasis-initiating cells (MICs) Figure 5 (H&E), as they always appear before the onset of frank malignancy i.e. as early as few weeks onwards until a full-fledged tumor is formed in the lung. These cells proliferate and have a high rate of apoptosis [3]. Interestingly when C-RAF is introduced with c-MYC as in MYC-BxB-DsRed model, there still exist the presence of pleomorphic cell clusters or better called as cryptic c-MYC transformants. However, in this case, C-RAF rescues these pleomorphic cell clusters from undergoing apoptosis hence rendering the model with accelerated tumor development [3]. In order to characterize these potential premalignant lesions and their lineage, expression of genes regulating proliferation and apoptosis were analyzed. Paraffin-embedded lung sections from MYC-DsRed and MYC-BxB-DsRed transgenic mice (> 12 months) were immunostained with c-MYC, cleaved caspase 3, Ki 67 and DsRed antibodies (Figure 5). These pleomorphic clusters exhibited strong nuclear c-MYC. Pleomorphic cells proliferate as seen by the Ki 67 staining and simultaneously exhibit cells undergoing apoptosis as observed by the presence of cleaved caspase 3 positive cells. Lineage marker for alveolar type II pneumocytes is maintained in the c-MYC positive foci as depicted by the expression of DsRed. The presence of DsRed in the clusters strongly suggests that they are alveolar type II cell-derived and have the potential to metastasize under the prolonged influence of the driver oncogene c-MYC.

### Lineage tracing captures early events of metastasis in the liver

**Figure 6:**
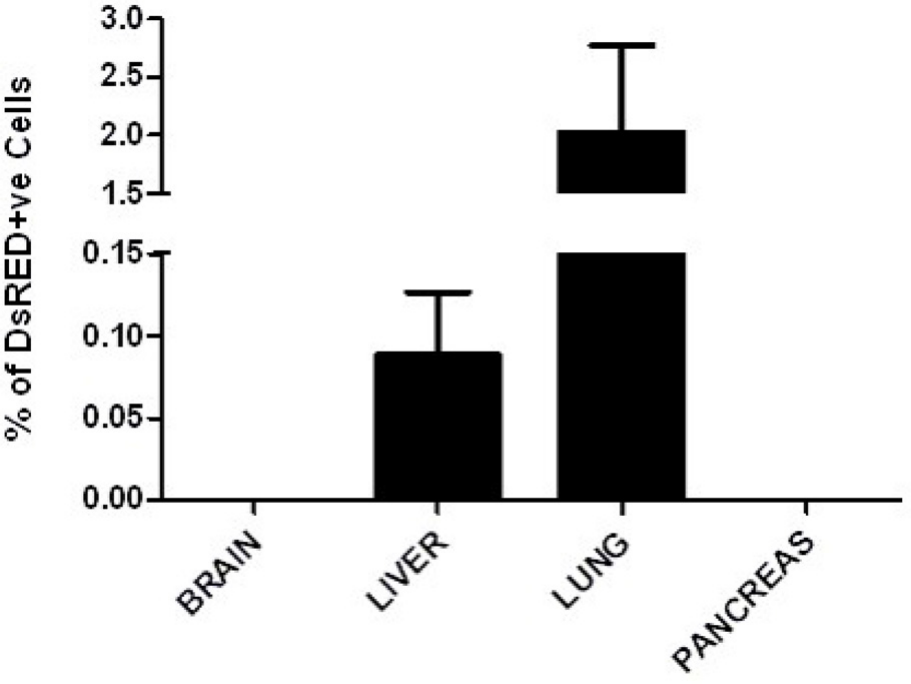
DsRed cells detected by flow cytometry is indicative of micro-metastasis Flow cytometry analysis of the different organs from the MYC-DsRed animals showed the presence of DsRed positive cells in the lung and liver only. The detection of DsRed in the liver indicates micro-metastasis as these animals did not show any visible tumors in the lung or liver. Data represents the average of three animals.

Previously in order to detect a micro-metastasis in the unlabeled single transgenic SPC-c-MYC and double transgenic SPC-C-RAF BxB/SPC-c-MYC mice, we used to do histological sectioning of the entire tissue from mice, and do an immunostaining for the lung progenitor markers. However, the greatest advantage of the current mouse model for metastasis is the incorporation of a fluorescent protein DsRed that allowed us to screen the presence of DsRed positive cells in different organs using a flow cytometry approach. Distant organs from metastatic mice MYC-DsRed were employed for FACS analysis. We chose those animals where no obvious visible metastasis and lung tumors were detected. Single cell suspension was created from the whole lung, liver, brain and pancreas from three MYC-DsRed mice which were between 10 to 14 months of age and were screened for the DsRed under the FLH-2 channel of the BD FACS caliber. Organs from the wild-type C57BL/*6* mice and the non-labeled SPC-c-MYC transgenic mice served as controls for this experiment. Interestingly, we found some fraction of cells positive for DsRed expression in the lung and liver of the MYC-DsRed transgenic mice, whereas brain and pancreas were found to be negative (Figure 6). This strongly suggests that micro-metastasis has already occurred in these mice, also indicating that it is an early event during the progression of metastasis in the liver large enough to be visible by naked eyes.

**Figure 7:**
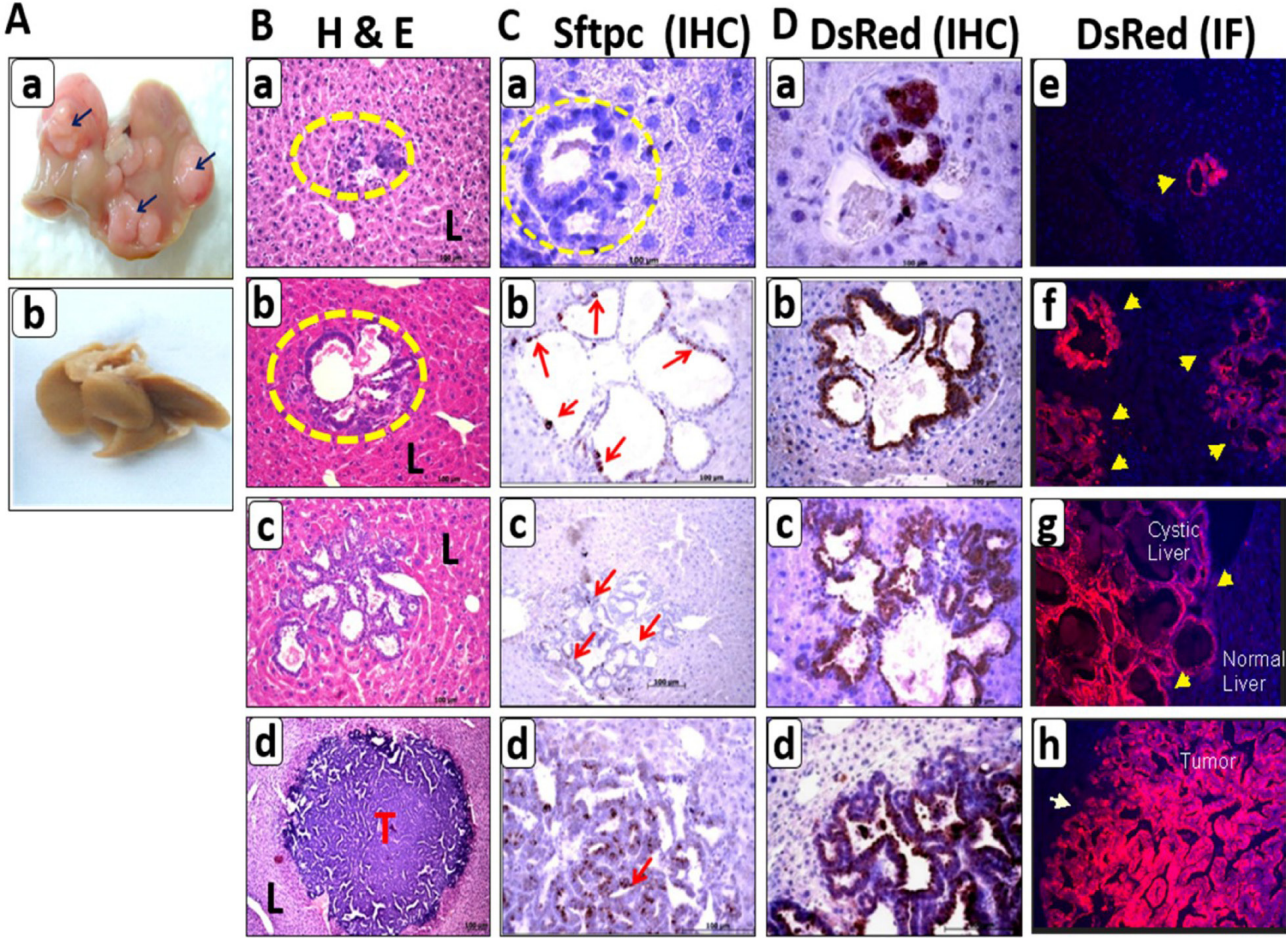
Lineage tracing in the metastatic model of Myc-BxB-DsRed transgenic mice depicts various steps of metastatic progression (**A**) Macrometastases, as seen in whole liver photographs, were developed in the liver of (a) a MYC-BxB-DsRed mouse (17 months induced) showing macroscopic tumor nodules on the liver (arrows) compared to (b) a non–induced mouse where the liver is clean/tumor-free. (**B**) Histopathological analysis of the liver metastases by H&E staining on paraffin-embedded liver sections shows the presence of three types of metastatic lesions: cystic (b), mixed cystic papillary (c) and papillary metastases (d). Small clusters of foreign cells are found streaming in the liver (yellow circles) (a). (T = tumor and L = liver). Scale bar = 100 μm. (**C**) Paraffin-embedded liver metastases immunostained for SPC showing Sftpc expression in cystic (b & c) and papillary metastasis (d). Small cell clusters are negative for the Sftpc expression (a) (yellow circles). Scale bar =100 μm. (**D**) DsRed immunostaining shows DsRed expression in all the subtypes of metastatic lesions beginning from small cell clusters (a), cystic (b), mixed cystic papillary (c) and papillary lesions (d). DsRed immunofluorescence on consecutive frozen sections also depicts the same pattern of DsRed expression (e, f, g, h). Arrows indicate the lesions positive for DsRed. Magnification 10X. Data is the representative images from four MYC-BxB-DsRed mice.

After having determined the expression of DsRed in the primary tumor (Figure 3A), our goal was to trace the fate of DsRed labeled cells in the metastatic colonization. We examined mice for metastasis after 12 to 15 months of induction. None of the C-RAF BxB-DsRed mice developed metastasis. However, multiple macroscopic liver metastases were observed in the MYC-BxB-DsRed mice [Figure 7A (a)]. On histo-pathological examination of the liver metastases, predominantly three types of structures were found. These were the cystic, mixed-cystic papillary and papillary lesions (Figure 7B).

Morphologically, the central portion of the cysts contained empty vacuole-like gaps and tumor cells were found predominantly outside the well-developed fibrous capsules in the liver [Figure 7B (b)]. While the cystic metastases have an intact periphery; papillary projections were usually recognized within the cystic lesions of the mixed cystic papillary metastases [Figure 7B (c)]. Papillary metastases have condensed cellular architecture with compact tumor cells composed of columnar cells, resembling that of the primary tumor [Figure 7B (d)]. Interestingly streaming single cells and clusters of cells were found scattered in the liver, indicative of initial seeds in metastatic colonization [Figure 7B (a)]. To facilitate the tracking of both lineage tagged Sftpc derived cells *in vivo* and DsRed positive tumor cells, metastatic lesions were immunostained with Sftpc and DsRed antibodies respectively (Figure 7C and 7D). Notably, small cell clusters were negative for Sftpc [Figure 7C (a)] and the first expression of few Sftpc positive cells was observed only in the cystic metastasis [Figure 7C (b, c)]. The number of Sftpc expressing cells increased in the mixed cystic papillary metastases and advanced in the papillary metastases as abundant Sftpc positive cells were present in them [Figure 7C (d)].

Strikingly, interesting observations were made by analyzing the distinct pattern of DsRed expressing cells in the metastases. Small cell clusters strongly expressed DsRed [Figure 7D (a)]. Cells contributing to cystic, mixed cystic papillary and papillary metastases were all positive for DsRed [Figure 7D (b, c, d)]. To rule out the possible artifacts related to DAB color development in the DsRed IHC, similarly, frozen sections were immunostained with the DsRed antibody. Immunofluorescence results depicted the same pattern of DsRed expression in metastatic lesions as with IHC [Figure 7D (e, f, g, h)].

**Figure 8:**
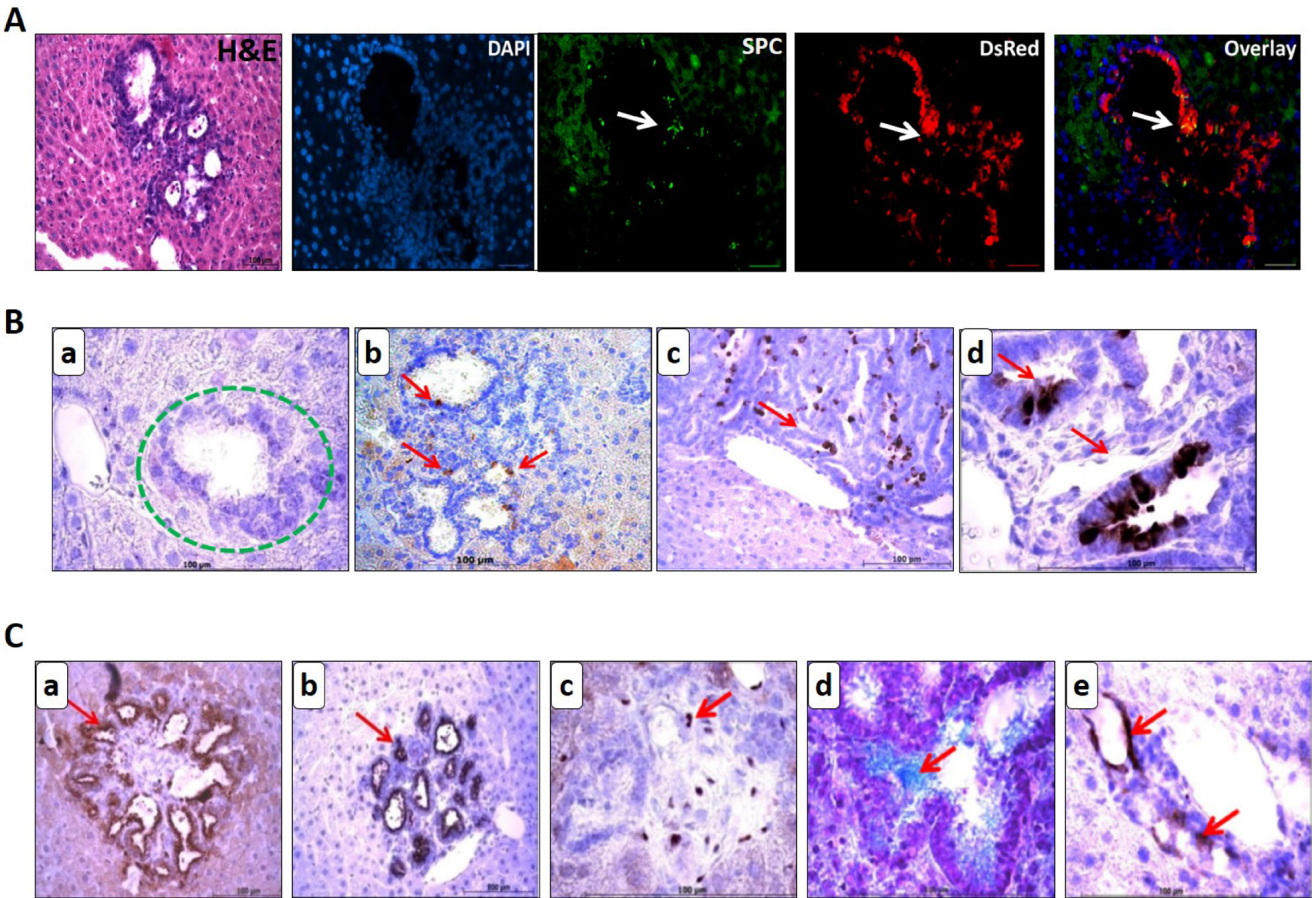
Lineage tracing reveals cystic lesions as metastasis in the liver and liver metastasis express the markers of pulmonary lineage (**A**) Cells in the cystic lesions that are negative for SPC expression are still positive for DsRed. Cryo embedded liver metastases sections were co-stained with SPC and DsRed antibodies. Few Sftpc positive cells (white arrow) appear near the papillary projections of a cystic metastasis which is strongly positive for DsRed. Scale bar = 100 μm. (**B**) Analysis of liver metastases for Clara cell marker via Scgb1a1 IHC. Image (d) is the higher magnification of (c) depicting the columnar shape of tumor cells in papillary metastasis positive for Scgb1a1. Scale bar = 100 μm. (**C**) Immuno-staining on paraffin embedded liver metastases sections reveals the presence of Pan-cytokeratin (a), Aquaporin 5, a marker for type I cells (b), and GATA 4 transcription factor (c). Mucin, a target gene for GATA 4 is also expressed in Alcian blue positive cells which mark the mucin positive cells (d) and neuro-endocrine marker PGP 9.5 was present in few cells of the metastases (e). Scale bar = 100 μm.

Cystic lesions being negative for Sftpc expression yet retaining the DsRed label, undoubtedly qualifies it to be metastatic lesion derived from alveolar type II cell precursors. Double staining for Sftpc and DsRed on cystic lesions shows DsRed cells that haven’t gained any Sftpc expression yet, while only one or two cells positive for Sftpc has been seen [Figure 8A].

Since a fraction of primary tumors exhibits the columnar cell phenotype, displaying the participation of non-ciliated columnar Clara cells in tumor formation, we analyzed the liver metastases for Clara cell marker, Scgb1a1 (Figure 8B). Small cell clusters and cystic metastasis were devoid of Scgb1a1 expression [Figure 8B (a) encircled green]. Few Scgb1a1 expressing cells were detected in the mixed cystic papillary metastases [Figure 8B (b)] and their number showed a pronounced increase in the papillary metastases [Figure 8B (c, d)]. This clearly indicates that Clara cells emerge late during the metastatic progression and thereafter populate the secondary tumors. We evaluated additional progenitor markers/lineage selectors for their expression in the metastases (Figure 8C). Candidate markers were selected based on their role during lung development. Liver metastases retained heterogeneous expression of pan-cytokeratin, aquaporin 5, transcription factor GATA 4 and its target gene Mucin. PGP 9.5 which was identified as a novel marker for tumor vasculature in mouse NSCLC [3] was highly expressed in the vasculature of liver metastases. These markers may, therefore, be suitable for early detection of metastases of NSCLC.

**Figure 9:**
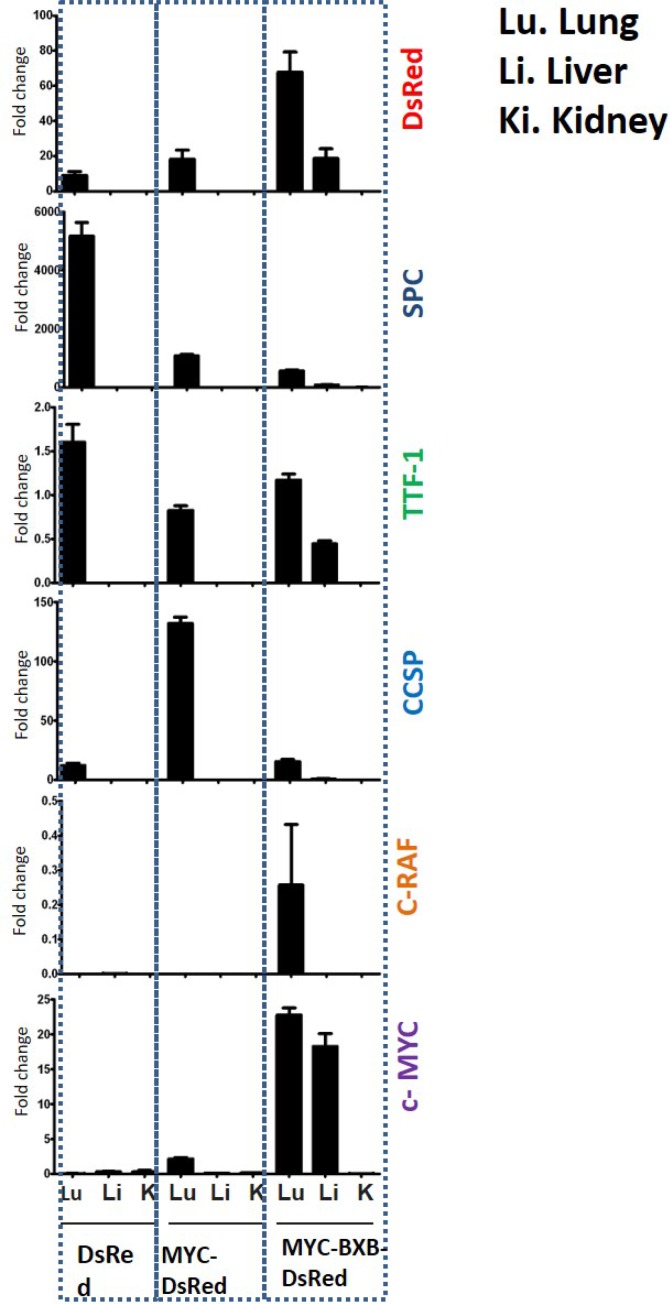
Validation of metastasis at the gene expression levels shows the expression of lineage-specific markers in the lung and liver of the metastatic mice MYC-BxB-DsRed mice Real-time PCR analysis showing the relative mRNA levels of C-RAF BxB, c-MYC, DsRed, SPC, CCSP, and TTF-1 genes in the lung, liver, and kidney of the DsRed, MYC-DsRed and MYC-BxB-DsRed mice. *n =* 3 +/– S.D.

Additionally, to validate the metastasis we analyzed the lineage specific markers in the lung, liver, and kidney of the MYC-BxB-DsRed metastatic mice at the gene expression level. mRNA levels of DsRed, SPC, c-MYC, C-RAF, and TTF-1 genes were analyzed by multigene real-time PCR using the cDNA obtained from the lung, liver, and kidneys isolated from DsRed, Myc-DsRed and MYC-BxB-DsRed transgenic mice. The non-neoplastic DsRed mice served as a control for this assay. Probes were normalized with HPRT as an internal housekeeping gene. Both the primary lung (with tumors) and its corresponding liver metastatic tissue displayed the expression of DsRed, SPC, c-MYC, and TTF-1 in the MYC-BxB-DsRed mice (Figure 9). This again suggests the expression of signature markers of the lung in the primary tumor as well as in the metastasis. Particularly, DsRed expression in the liver metastasis further strengthens the fact the alveolar type II cells are one among the other cell types that contribute to the metastasis of NSCLC. We do not deny the fact that there might be other important cell types of the lung yet undiscovered that are implicated in the NSCLC metastasis. Through the current report, we validated the identity of cystic lesions and demonstrated that alveolar type II cells are involved in mediating the metastasis of NSCLC in mice with c- MYC in conjunction with C-RAF as the driver oncogenes.

## DISCUSSION

Lung cancer is the leading cause of cancer deaths worldwide [2] and adenocarcinoma is currently the most common type of lung cancer. Cell turnover in the adult lung is normally very low [17]. However, epithelial injury elicits rapid proliferation of surviving cells and the tissue is soon repaired. Studies suggest that alveolar epithelium is repaired by the alveolar type II cells, hence serving as the progenitor cells [18]. Different studies and approaches including lineage tracing have given important information regarding different cell populations that act as progenitor cells of the lung under distinct circumstances and niches.

For e.g. Basal cells in the proximal airways, are known to act as stem cells in the bronchiolar epithelium [19]. Cells located at the bronchiolar-alveolar duct junction (BADJ) are identified as bronchioalveolar stem cells (BASCs) [20]. Also, calcitonin gene-regulated peptide (CGRP)-expressing neuroendocrine cells have been reported to self-renew and differentiate into ciliated and Clara cells after naphthalene injury [21]. Another cell population having CD45^neg^ CD31^neg^ EpCAM^high^ integrin α6pos CD104^pos^ CD24^low^ signature [22] and integrin α6β4-positive cells [23] residing in the alveolar epithelium are identified as progenitor cells of the airways. Most recent studies indicate that alveolar type II cells switch their function as stem cells thereby contributing towards the renewal of lung cells, repair and in neoplasias [24].

Despite the evidence that alveolar type II cells are the progenitor cells of the alveolar epithelium compartment; relatively little is known about their biology and the mechanisms regulating their behavior and participation in the metastatic progression of malignant tumors. Mouse models serve as an appropriate tool to study individual cell populations for their capacity to give rise to distinct tumors [25]. Such approach has been nicely demonstrated in pancreatic cancer models using cell type-specific Cre expression [26, 27]. The current study provides an *in vivo* description of an adult proliferative differentiated cell compartment (i.e. the alveolar epithelium) where we have observed a specific population, identified by DsRed expression in the liver metastasis that verified the contribution of alveolar type II cells in the metastatic colonization in NSCLC. Expression of DsRed in the lung tumors functioned as a readout for Sftpc expressing alveolar type II cells. This supports the presence of a Sftpc positive progenitor cell population that can give rise to NSCLC following overexpression of c-MYC and C-RAF.

The presence of DsRed positive cells in the cystic metastasis suggests that these DsRed positive cell clusters have been originated from the Sftpc producing alveolar type II cells. This shows that the resulting metastases were clonal at this stage of cystic appearance, additionally suggesting that these cystic metastases should have resulted from the proliferation of a single viable DsRed positive cell (alveolar type II) within a circulating tumor embolus which is likely to be homogenous for DsRed expression. Interestingly, the presence of many purely cystic liver metastases expressing DsRed positive cells in the lining epithelium argues that cysts happen to be a transitional state to the development of frank malignancy. Hence with our lineage tracing data in NSCLC metastases, we propose that cysts are the precursors of advanced liver metastases.

**Figure 10:**
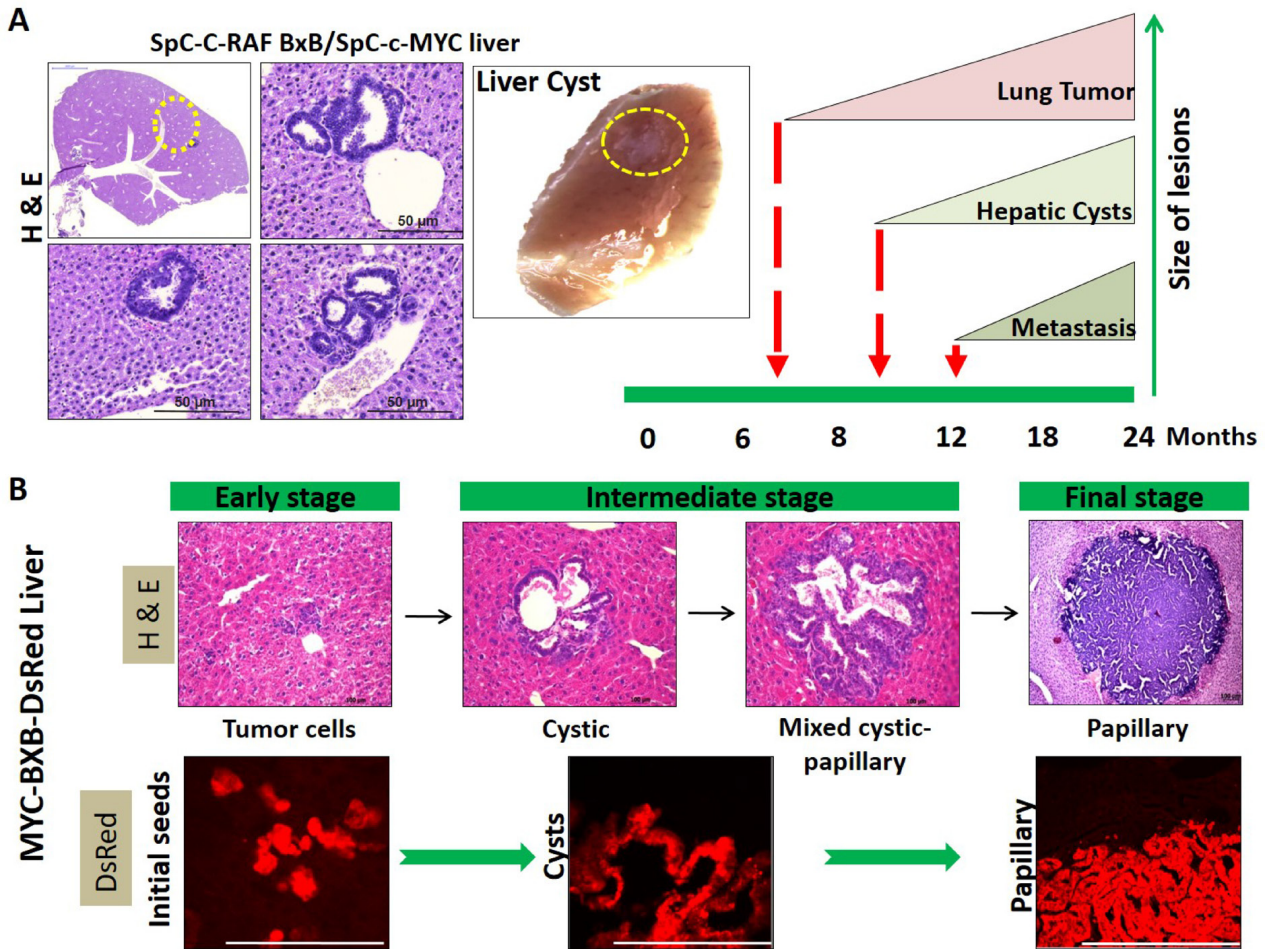
Hepatic cysts represent the early stage of metastasis in NSCLC as revealed by lineage tracing (**A**) Observing the single transgenic SPC-c-MYC and double transgenic SPC-C-RAF BxB/SPC-c-MYC mice for a period of two years displayed the occurrence of small cystic lesions and big papillary metastases in the liver. The appearance of cystic lesions starts from 6 months onwards, whereas the onset of frank metastases large enough to be visible by naked eyes occurs around 10 to 12 month onwards. H&E stained liver section of the SPC-C-RAF BxB/SPC-c-MYC mice showing cystic structures. The whole liver photograph depicts the small cystic lesion indicated in the yellow circle. (**B**) Cystic, mixed cystic papillary and papillary lesions exist together within the same tissue i.e. liver (H&E). With the help of lineage tracing; the occurrence of single DsRed positive cells streaming within the liver as well as DsRed cells comprising all the different types of lesions in the liver (DsRed fluorescence image), suggests that these hepatic lesions are indeed metastasis and cystic lesions are the first to be detected at an early time point much before the full fledge metastases are observed. This makes the cystic lesions to be the precursors of frank metastasis. Moreover, as papillary lesions being the largest in size and cystic being the smallest suggests that the cystic lesions advance with time and finally progress to papillary metastases. Paraffin-embedded metastatic liver sections stained with H&E. Corresponding cryo metastatic liver sections observed under the RFP channel shows DsRed fluorescence.

We have frequently observed small cystic lesions in the liver of the non-labeled single transgenic SPC-c-MYC and double transgenic SPC-C-RAF BxB/SPC-c-MYC mice (Supplementary Figure 2). These lesions were detected as early as six months of age. But we were unable to determine the identity of these lesions, due to the lack of a suitable marker. However, in the current model with the introduction of DsRed in the transgenic SPC-c-MYC and SPC-C-RAF BxB/SPC-c-MYC mice, we have shown that these cystic lesions are metastasis and represent an early stage of the malignant progression. The occurrence of liver metastasis comprising dozens of individual tumor foci provides a strong view that MICs have spread at a very early stage of the lung tumor development, perhaps during the time when the primary tumor in the lung was being formed. This notion is supported by the fact that we observed small DsRed cell clusters streaming in the liver. Moreover, the existence of all the three different metastasis lesions together i.e. cystic, mixed cystic and papillary along with individual DsRed cells in distant sites strongly supports the theory that metastasis is a continuous process and indicates that cysts may be a transitional state to the development of malignancy (Figure 10).

The very fact that metastatic lesions in the liver and the primary tumor in the lung share markers of alveolar lineage DsRed and markers of alveolar type I cell i.e. aquaporin 5; indicates that aggressive malignant cell progenies are derivatives of the parental primary tumor. This goes in accordance with the fact that metastasis and primary tumors share somatic mutations. In view of this, the assumption that primary and metastatic tumors have common ancestry is inherent in the design of systemic treatment strategies for advanced cancers. Important examples include *HER2* amplification in breast cancers and their subsequent lymph node or distant organ metastasis [28]. Metastatic lesions express Sftpc, GATA4 transcription factor and its direct target gene Mucin as evident by the Alcian blue-positive cells in the lesions. The expression of these early progenitor markers within the metastases strongly supports the existence of cells; characteristics of early cancer precursors. Non-malignant lesions have been sporadically observed in the livers of many cancer patients in various stages of tumor progression [29–31].

In our mouse model of NSCLC [SPC-c-MYC and SPC-C-RAF BxB/SPC-c-MYC [3]], we consistently observed cystic lesions along with malignant lung tumors that have metastasized to liver and other distant organs. Moreover, occasionally early lesions were also observed sometimes in a liver where no obvious metastasis of lung tumor has occurred yet. Although we speculated these lesions might represent early stages of metastasis seeding, they did not express any lung differentiation markers except very few cells positive for Sftpc in the cystic lesions. However, in the present study, we were able to show that the DsRed reporter gene is activated in the SPC producing type II cells and subsequently transmitted to their progeny as they repopulate the tissue during normal homeostasis (in non-neoplastic, DsRed animals) and in tumor formation. Because the DsRed labeled, Sfptc positive type II cells are progenitors of the alveolar epithelium, they would continuously generate DsRed progeny, which contributes to tissue renewal over the entire lifetime of the mouse and would also exhibit its expression in the very late stages of abnormalities as in the case of metastasis formation. Thus, using lineage tracing approach in the current report, we prove that cystic lesions in liver mark the early stage of metastatic invasion which can have significant implications for early diagnosis of NSCLC metastasis in clinical settings.

### Perspective

**Figure 11:**
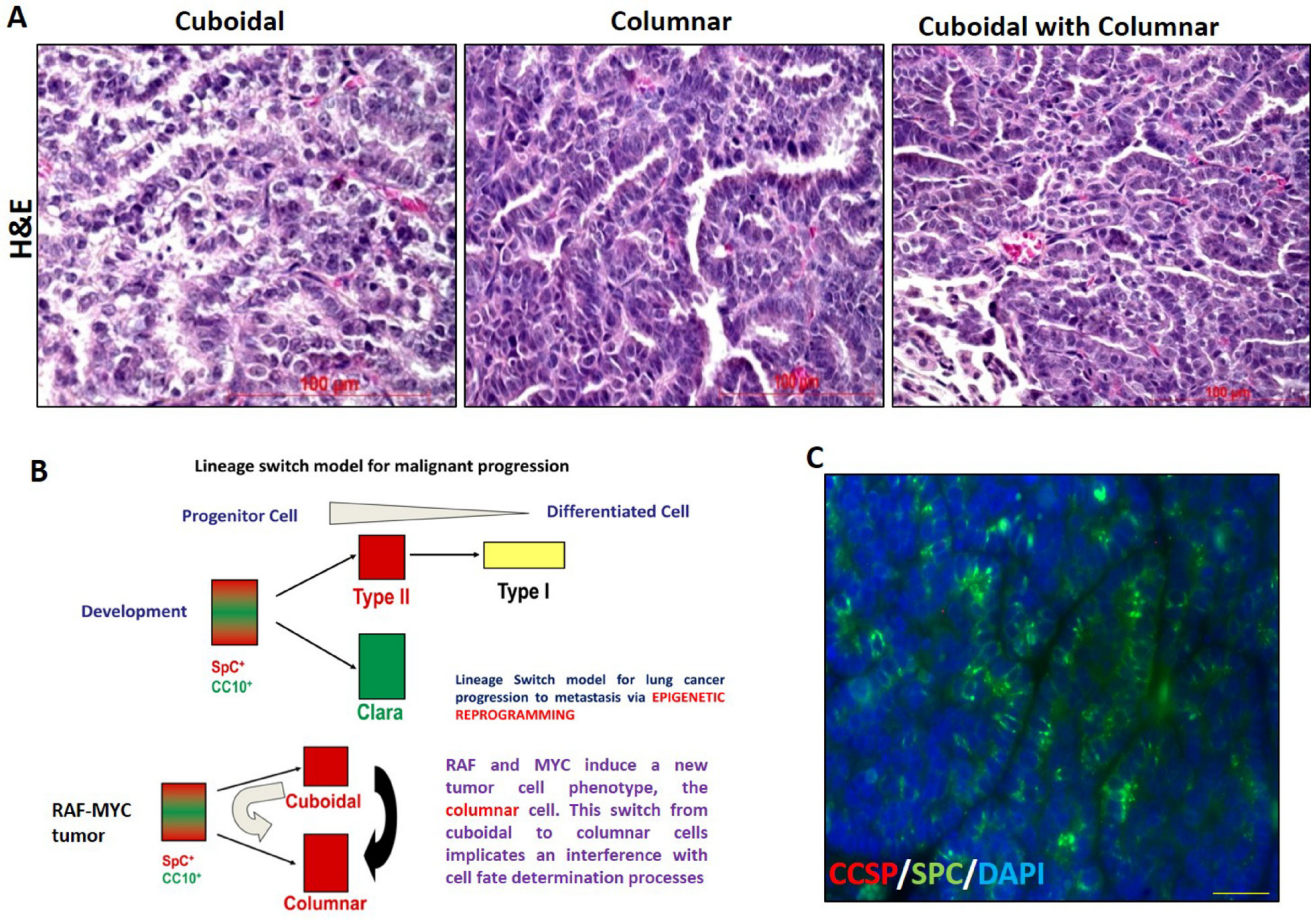
Proposed lineage switch model for malignant progression in NSCLC depicting the differential morphology gained by type II cell during tumor progression (**A**) H and E staining of tumor showing cuboidal, columnar, and mixed cuboidal with a columnar morphology of tumor cells derived from C-RAF BxB-DsRed, MYC-DsRed, and MYC-BxB-DsRed mice respectively. (**B**) Proposed model suggest the existence of a common multi-potent progenitor cell that exhibits a phenotype which expresses both SPC and CCSP and finally differentiates into type II cells and Clara cells during the normal development of the pulmonary epithelium. But in the case of tumorigenesis, with the introduction of c-MYC, in particular, shows a different program where c-MYC interferes with the normal cell fate determination process thereby reprogramming the progenitor cells in the tumor to acquire columnar cell morphology, yet producing SPC as seen in (C). (**C**) Double Immunofluorescence staining of a MYC-DsRed lung tumor shows tumor cells with a columnar morphology that are negative for CCSP, yet expressing SPC.

The distinct morphology of tumor cells i.e. the cuboidal and columnar driven by the distinct oncogenes, i.e. the C-RAF and c-MYC respectively (Figure 11A); suggest the existence of a unique pathway in the development of adenoma, where tumor arises from a cell type that has characteristics of both the Clara cells and type II cells. This unique pathway can be described by two possible models (Figure 11B).

The first model suggests that the activation of oncogene induces the hyper-proliferation of Clara cells that further undergoes the trans-differentiation process as the cells move into the alveolar compartment. However, an alternate model is also suggestive of the fact that the existence of few stem cells with K-Ras activation may result in the production of those intermediate cell types that have the potential of developing either into Clara cells or alveolar type II cells. Hyperplasia of such intermediate cell might be the first stage of tumorigenesis that can give rise to adenoma at a later time. This notion is supported by the fact that we have detected a fraction of NSCLC tumors expressing CCSP, which further suggests that CCSP is expressed in the lineage of the cell of origin in NSCLC. This also indicates the existence of an early progenitor cell that in spite of expressing a low level of CCSP has the capacity to differentiate along the alveolar lineage. This cell could either be a common progenitor which resides in the lung or a committed progenitor having the potential to transdifferentiate under the pressure of the concomitant expression of c-MYC. Previous studies by other investigators reporting the evidence for a multi-potent progenitor cell in the immature and developing lung [32, 33] is supportive of our present hypothesis claiming the presence of a multi-potent progenitor. Speaking about the driving oncogenes in our metastasis versus non-metastasis model, one cannot ignore the influence of the specific oncogenes on the target cell. In view of this, it is important to mention that c-MYC is a powerful reprogramming factor which critically controls the cell differentiation process, thereby inducing a lineage switch in the cell fate determination process. Hence it is an important factor determining the normal and cancer cell differentiation [34]. Therefore, c-MYC overexpression could be a mechanism by which a progenitor-like alveolar type II cell has the propensity to differentiate towards a more neuro-epithelial and bronchiolar cell-like state (Figure 11B). Thus one cannot ignore here the plasticity of the type II cells and its behavior that is ultimately determined by the oncogene [9]. In view of this, we have also detected tumor cells in the lung that have columnar morphology negative for Clara cell marker CCSP, yet positive for SPC (Figure 11C). This shows that tumor cells acquire a columnar morphology and forms a major cell mass of the papillary tumors, being negative for CCSP, still expressing SPC. A recent study shows that the cell of origin in lung adenocarcinoma exerts its influential effect on the histopathological phenotypes of the resulting tumors [35].

**Figure 12:**
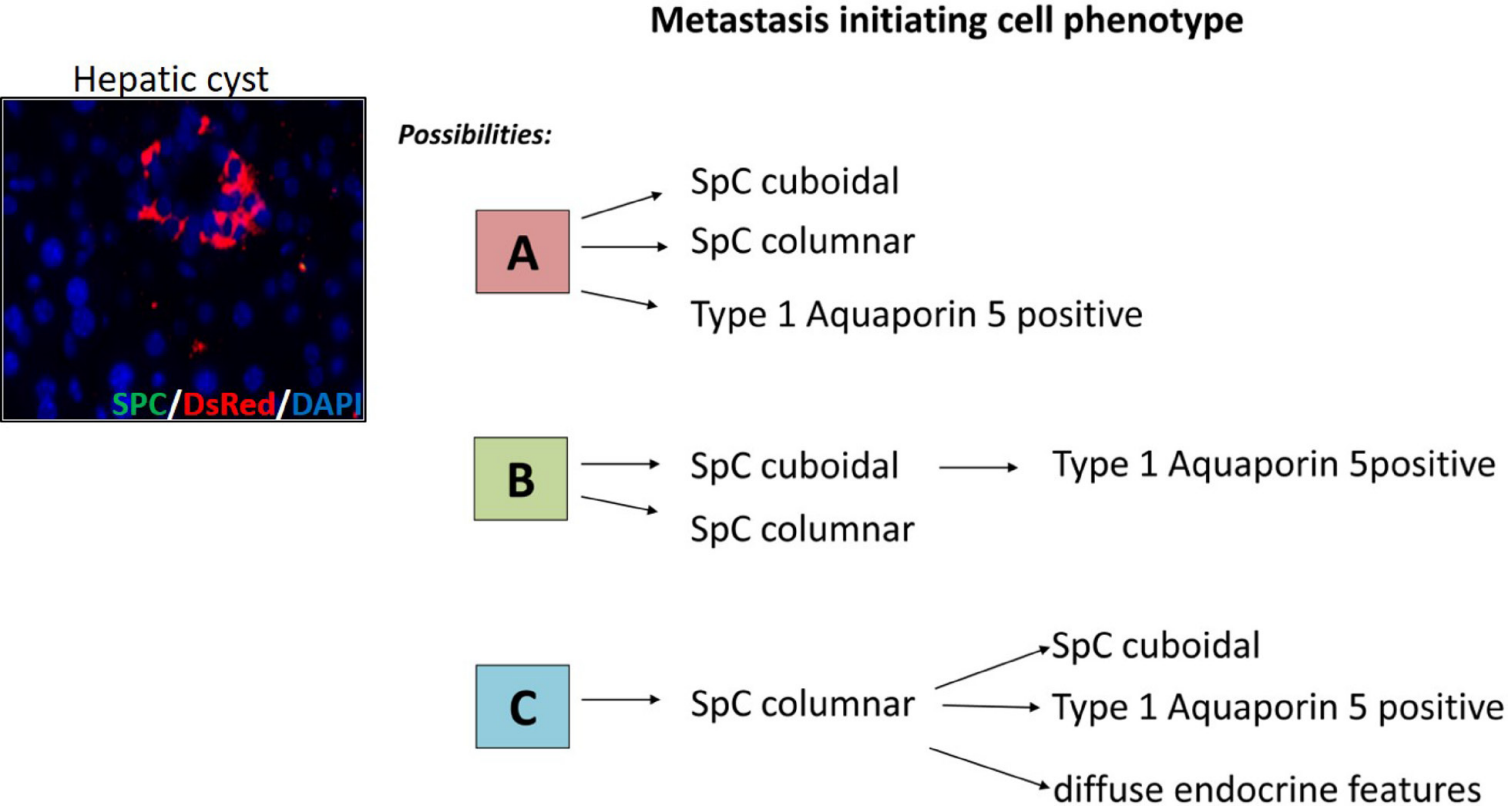
The proposed identity of the putative cell types responsible for metastasis in the current NSCLC model The cells comprising the cystic lesions of the hepatic cysts are positive for DsRed (SPC/DsRed Immunofluorescence), yet no SPC is expressed at this stage clearly demonstrates that these DsRed positive cells were derived from an SPC producing type II cell lineage. Based on this we propose the participation of an SPC-expressing progenitor cell type in the metastatic colonization. This progenitor cell will have three kinds of phenotypic possibilities which it acquires as shown by the putative identities of cell A, B, and C.

With the lineage tracing data, we were able to demonstrate that DsRed positive cells i.e. the SPC-positive alveolar type II cell lineage, in the hepatic cysts marks the early stage of metastasis in NSCLC. DsRed labeled cells are the readout for SPC producing type II cells and are considered as the progenitors of the alveolar epithelium. To further conclude the contributory role of alveolar lineage in determining the characteristic of metastatic initiating cells, our data supports the existence of at least three different progenitor cell pools in the pulmonary epithelium, which acts as a putative metastatic initiating cell (Figure 12).

The first compartment comprises of an actively proliferating stem cell that is predominately responsible for the daily maintenance of the alveolar and bronchiolar epithelium and is termed as bronchioalveolar stem cells (BASCs). The fact that cancer stem cells are rare, resistant to therapy and hence responsible for cancer relapse [36]; supports the notion that cancer stem cells in the lung can be one of the sources of metastasis. In accordance with this assumption, we detected few BASCs in the primary lung tumor of the MYC-BxB-DsRed mice (Supplementary Figure 1).

The second compartment comprises of the SPC-expressing type II cells with a cuboidal morphology which acquires a columnar phenotype at a later time point. Once these cells attain the columnar shape resembling like bronchiolar structure, they presumably start producing CCSP to sustain the bronchiolar epithelium lineage. Notably, in our mouse model, the tumor cells fueled by C-RAF (C-RAF BxB-DsRed) hold a cuboidal morphology whereas the tumors driven by c-MYC (MYC-DsRed) shows columnar cell morphology, which is comparatively larger in size as well as thought to be having a more aggressive phenotype than the cuboidal ones. These are also called as the papillary tumors and are generally observed at an advanced stage of the tumor progression as we have observed this phenomenon in our previous investigations [3] further strengthening our hypothesis.

The third compartment belongs to the SPC-expressing columnar type II cells that later acquire a cuboidal phenotype and gain some neuroendocrine features (Figure 12).

However, metastasis being complex phenomena depends upon the interplay of several factors like the initial transforming mutation in the subpopulation of a primary tumor, the genetic background of the organism and the cell type from which the tumor originates and most importantly the tumor-microenvironment. Essentially, in most cases, none of these factors alone are the sole determinants of metastasis development in an organism. Nevertheless, with the help of genetic lineage tracing approach in an NSCLC model, we have proved that alveolar type II cells are the crucial cell type responsible for metastasis and that hepatic cysts mark the very early stage of metastasis.

## MATERIALS AND METHODS

### Transgenic animals

This study was carried out in strict accordance with the recommendations and regulations of the Bavarian State authorities. The protocol was approved by the Regierung von Oberbayern, 80538 München (Permit Number: 55.2-1-54-2532-18-11) and Regierungspräsidium Darmstadt, 64283 Darmstadt (Permit Number: V 54-19c 20/15-B2/233) Germany. Mice were euthanized with a lethal intraperitoneal injection of Ketamin and Rompun in 0.9% NaCl, anesthesia and all efforts were made to minimize suffering.

Mice were maintained as a specific pathogen-free in-house colony. Mice were allowed food and water ad libitum and maintained on a 12-hour/day light/dark cycle. Mice harboring the Lac Z floxed-DsRed recombination substrate (RS) transgene on a C57BL/6J background were purchased from The Jackson Laboratory and maintained by mating hemizygous individuals. The generation of SPC-C-RAF BxB transgenic mice has been previously described [14]. SPC-c-MYC mice express the murine oncogene c-MYC under the control of lung-specific surfactant protein C promoter. DsRed, SPC-rtTA, SPC-C-RAF BxB and SPC-c-MYC mice were routinely bred in the C57BL/6 background whereas TetO-Cre animals were kept in the FVB/N background. DsRed mice were mated with SPC-rtTA and TetO-Cre mice to obtain triple transgenic SPC rtTA/TetO-Cre/DsRed (hereafter DsRed). Triple transgenic mice were mated with SPC-C-RAF BxB and SPC-c-MYC mice mice to obtain quadruple transgenic animals SPCrtTA/TetO-Cre /TetO-C-RAF BxB/DsRed (Hereafter C-RAF BxB-DsRed) and SPCrtTA/TetO-Cre /SPC-c-MYC/DsRed (hereafter MYC-DsRed) respectively. Quadruple transgenic mice were mated with SPC-C-RAF BxB or SPC-c-MYC to obtain penta transgenic as SPCrtTA/TetO-Cre/TetO-C-RAF-BxB/SPC- c-MYC/DsRed (hereafter MYC-BxB-DsRed). DsRed expression in normal and in tumor-bearing lungs was carried out by induction of compound mice with doxycyclin (DOX) (Sigma) containing food (500 mg/kg body weight; Ssniff, Soest, Germany) throughout gestation and until euthanization. Animals used in this study were from 2 to 24 months old. Animals were sacrificed via cervical dislocation unless described differently.

### Genotyping

Mouse tails were cut (less than 1 cm) during the age of 3 to 4 weeks. The tails were lysed by the addition of 190 µl of tail lysis buffer and 12 µl of 0.4 mg/ml Proteinase K and incubated overnight at 54°C. The resulting lysate was then centrifuged at 10000 rpm for 15 minutes and the resulting supernatant was diluted 1:10 or 1:40 with distilled/autoclaved water. This was used as a template for a PCR reaction with specific primers for genotyping. In some cases, a preliminary step of DNA isolation followed by PCR detection was necessary. Transgene positive mice were identified by PCR analysis of tail DNA using primers specific for the LacZ, DsRed, SPCrtTA, Tet O Cre, Tet O C-RAF BxB, and SPC-c-MYC coding sequences.

### Histology and immuno-staining

Animals were sacrificed and perfused with phosphate buffer saline (PBS). Lungs and liver were dissected and fixed under 25 cm water pressure with 4 % buffered paraformaldehyde and paraffin embedded (Shandon Citadel 2000). Tissue blocks were sectioned to 6 µm sections by using a manual rotary microtome (Leica RM2235). For Optimal cutting temperature (OCT)-embedding and cryosectioning, fresh tissue was washed with PBS and fixed in 4% PFA in PBS at 4°C overnight, transferred into 30% sucrose in PBS and incubated at 4°C until the tissue falls to the bottom of the tube owing to density. Tissues were embedded on dry ice with OCT (Tissue-Tek) and blocks were sectioned into 6–10 µm and stored at –80°C until use. For histological analysis of paraffin-embedded tissues, sections were deparaffinized in xylene, rehydrated in graded series of alcohol and stained with Hematoxylin and Eosin. For immunohistochemical stainings, sections were deparaffinized and rehydrated. Heat-mediated antigen retrieval was performed by boiling the sections in 10 mM Sodium citrate buffer, pH 6 for 10 to 20 minutes in a microwave. Endogenous peroxidase activity was quenched by incubating the tissue slides with 1.5–3% H_2_O_2_ in PBS or methanol for 30 min at room temperature. To block the non-specific binding of immunoglobulin, slides were incubated with a blocking solution consisting 5% rabbit, goat or donkey serum and 0.2% triton-X 100 in PBS for one hour at room temperature followed by incubation with the primary antibodies diluted in the blocking solution overnight at 4°C. Subsequently, biotinylated secondary antibodies (DAKO Denmark) was applied at 1:200 dilution and incubated for 1–2 hours at room temperature. The slides were then incubated with an ABC reagent (Vectastatin Elite ABC kit, Vector Labs) for 45 min at room temperature and the chromogen was developed with DAB (0.8% H_2_O_2_ in 1 ml DAB). The slides were counterstained with hematoxylin, dehydrated and mounted with Entellan. The Omission of primary antibodies was used as a negative control in one slide from each staining series. All incubation steps were carried out in a humidified chamber and all washing steps were performed with PBS. For SPC/DsRed double immunofluorescence staining, all steps were almost similar except that Donkey anti-goat Cy5 and Donkey anti-rabbit Cy3 were used as secondary antibodies (Jackson ImmunoResearch). Sections were counterstained by 4′, 6- diamidino-2-phenylindole (DAPI) and mounted with Mowiol. DsRed (Clonetech) was applied on frozen section and for paraffin embedded sections, DsRed (Santacruz) was used. For Alcian Blue staining, slides were incubated with Alcian Blue 2.5 solution for 30 minutes at room temperature, washed under tap water to get rid of the excess stain and then incubated with Nuclear Fast Red solution for 3 to 5 minutes, washed, dehydrated and mounted. Images were captured and processed using AxioVision software (Carl Zeiss Microscopy GmbH).

### Flow cytometry

Mice were anesthetized with Ketamin/Rompun formulation and were cleaned by spraying 70% alcohol. An incision was made over the rib cage and 10 ml of cold saline solution was injected into the right ventricle until the lungs and other organs were cleared off from the blood. Lung, liver, and kidney were dissected out and immediately kept in a clean sterile petridish on the ice and observed for any visible metastasis. Thereafter the individual organs were placed in a 50 ml falcon tube containing 6 ml of PBS (sterile cell culture grade) in cold.

Enzymatic digestion of the whole organ was carried out by the addition of collagenase/dispase, and agitated for 45 minutes at 37°C on a shaker. After the incubation, the tissue was squeezed gently like a tea bag using forceps until it was turned into a mass of suspension, leaving behind only the connective tissue. At this stage, DNase I was added. The digested tissue was placed on ice for 5 minutes. Subsequently, the digested tissue suspension was filtered through 100 µm and then via 40 µm cell strainer to obtain a clear single cell suspension. The suspension was collected in a 50 ml falcon tube, centrifuged at 800 rpm for 8 minutes at 4°C. A soft pellet was obtained. The supernatant was discarded. The pellet was resuspended in 2 ml of FACS buffer and was proceeded for FACS analysis. DsRed screening was carried out in FLH-2 channel of the BD FACS, caliber.

### Real-time PCR

RNA isolation from tissues was performed using TRIzol Reagent (Invitrogen) according to the manufacturer’s instructions. The concentration of the isolated RNA was determined by measuring the absorbance at 260 nm (A260) using a spectrophotometer. Total RNA obtained from eukaryotic cells or tissue was treated with RNase–free DNaseI to remove the genomic DNA contamination from the RNA sample. The first strand synthesis was performed using the fermentas first strand synthesis kit and random hexamer primers as described in the instruction manual. 1 μg cDNA was used as a template for qPCR assay with 10 µl of Finnzymes SYBR Green master-mix, 0.5 µM of the primers specific for the gene of interest or for the house keeping gene, and 0.4 µl of the dye ROX as an internal control were used in a 20 µl reaction. To calculate the relative amount of the transcript of the gene of interest, the amplification efficiency was raised to the power of the threshold cycle (Ct –value). This gave the number of cycles necessary for the product to be detectable. The resulting value was normalized to the level of the house keeping gene for all samples in the same experiment. Assays were performed in triplicates following the manufacturer’s instructions in an Applied Biosystem set up.

### Reagents and antibodies

For immunohistochemistry and immunoflu-orescence staining, primary antibodies against the following proteins were used: Rabbit anti DsRed monoclonal (Clonetech), DsRed (L-18 Santa Cruz), CC10 (S-20 Santa Cruz), c-MYC (N-262 Santa Cruz), GATA-4 (C-20 Santacruz), PGP 9.5 (78-504 AbD Serotec), PCNA (555566 Becton Dickinson Co), pro SPC (Gift from J. Whitsett), Actin (I-19 Santacruz), Aquaporin 5 (Alomene Aqp-005),C-RAF (E 10) Santa Cruz), HA Tag (clone 3F10 Roche), Cleaved Caspase 3 (Cell Signaling).

### Statistical analysis

Statistical analyses were performed using the Graphpad Prism version 4.0 software (Graphpad Software, Inc, San Diego, CA). Differences among the groups were compared with Student’s *t*-test (two-tailed). *P* < 0.05 was considered significant. The statistical significance between proportions was determined by the log-rank test.

## SUPPLEMENTARY MATERIALS FIGURES



## Abbreviations

NSCLC: Non-small cell lung cancer
SCLC: Small cell lung cancer
SPC: Surfactant protein C, Sftpc
CCSP: Clara cell secretory protein, Scgb1a1
MAPK: Mitogen-activated protein kinase
PGP-9.5: protein gene product 9.5
IHC: Immunohistochemistry
DAB: Diaminobenzidine
MICs: Metastatic initiating cells
BASC: Bronchioalveolar Stem Cells
