# Supplementary material for: Cysts mark the early stage of metastatic tumor development in non-small cell lung cancer
Source: Oncotarget. 2017 Dec 30;9(5):6518–35. doi: 10.18632/oncotarget.23785 (PMC5814229; doi:10.18632/oncotarget.23785)
Supplement: Supplementary file 1 [file oncotarget-09-6518-s001.pdf]

Cysts mark the early stage of metastatic tumor development in non-small cell lung cancer

SUPPLEMENTARY MATERIALS

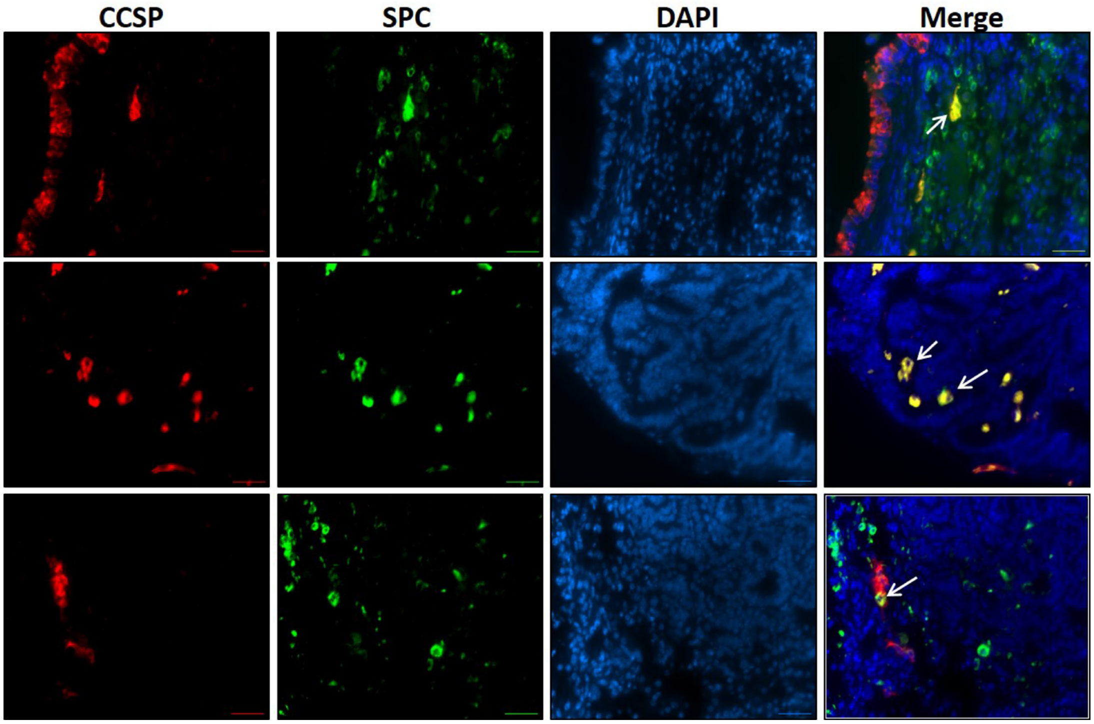

Supplementary Figure 1: Presence of circulating bronchioalveolar stem cells (BASCs) in the primary tumors of the MYC-BxB-DsRed mice Paraffin-embedded lung sections of the 12-month induced MYC-BxB-DsRed mice were co-immunostained with the SPC and DsRed antibodies. DAPI shows the nucleus. Arrows indicate the double positive BASCs. *N* = 2, Scale bar =100 μm.

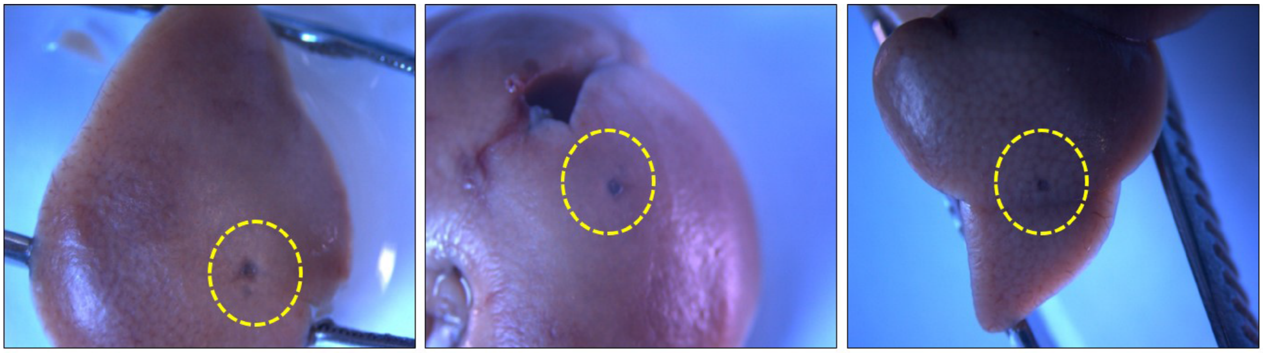

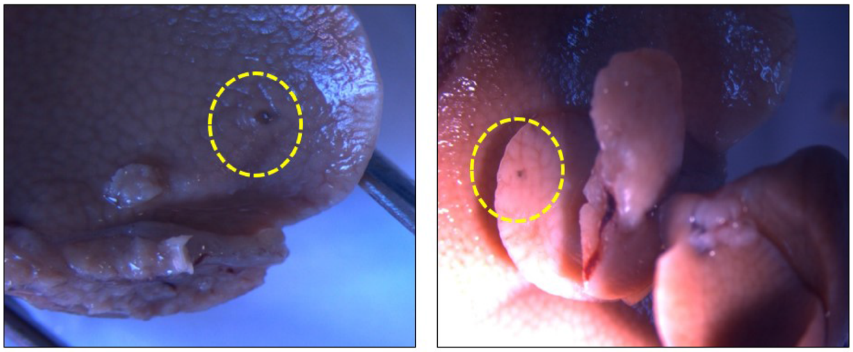

SPC-c-MYC

no. of animals	Months
1	10
1	10.75
1	13
1	13.75
1	14.25
2	14.5
1	16.75
2	17.5
1	18
1	18.75
1	20.5
1	22
1	26

SPC-C-RAF BxB/SPC-c-MYC

no. of animals	Months
1	10.75
2	12
2	12.75
1	13.5
1	13.75
1	14.5
1	15
4	15.5

Supplementary Figure 2: Appearance of cysts in the livers of unlabeled transgenic SPC-c-MYC and SPC-C-RAF BxB/SPC-c-MYC mice Representative photographs of whole liver derived from unlabeled single transgenic SPC-c-MYC and double transgenic SPC-C-RAF BxB/SPC-c-MYC mice. Mice were monitored and were sacrificed from 10 months onwards until 26 months, and showed the presence of hepatic cysts.
